# Improvement effect of compound Ento-PB on oxazolone-induced ulcerative colitis in rats

**DOI:** 10.1590/acb395524

**Published:** 2024-09-02

**Authors:** Zhi Fan, Jinhu Chen, Jia Wei, ZhiBin Yang, Huai Xiao, Heng Liu

**Affiliations:** 1 Dali University – Yunnan Provincial Key Laboratory of Entomological Biopharmaceutical – Dali – China.; 2 Dali University – National-Local Joint Engineering Research Center of Entomoceutics – Dali – China.; 3 Dali University – Engineering Research Center for Development and Comprehensive Utilization of Entomoceutics – Dali – China.

**Keywords:** Colitis, Ulcerative, Oxazolone, Anti-inflammatory Agents

## Abstract

**Purpose:**

To investigate the impact of the Chinese medicine compound Ento-PB on oxazolone (OXZ)-induced ulcerative colitis (UC) in rats.

**Methods:**

UC rats induced by OXZ were treated with Ento-PB. The damage to the colon was assessed using several measures, including the disease activity index (DAI), colon length, colon weight/length ratio, colonic mucosal damage index, and histological score. The levels of interleukin-4 (IL-4), interleukin-10 (IL-10), interleukin-13 (IL-13), epidermal growth factor (EGF), inducible nitric oxide synthase, and total nitric oxide synthase (tNOS) in rat serum, as well as the levels of tumor necrosis factor-α (TNF-α) and myeloperoxidase (MPO) in rat colon tissue, were determined using enzyme-linked immunosorbent assay and conventional kits.

**Results:**

After being treated with Ento-PB, the DAI score and macroscopic lesion score of OXZ-induced UC rats were significantly reduced. Ento-PB prevented the shortening of rat colons, reduced the ratio of colon weight to length, and improved colon tissue lesions. Meanwhile, Ento-PB could significantly inhibit the activities of proinflammatory cytokines TNF-α, IL-13, and MPO, as well as tNOS and iNOS, while upregulating the expression of anti-inflammatory cytokines IL-4 and IL-10. Moreover, a significant increase in the expression level of EGF was observed in UC rats treated with Ento-PB, indicating that Ento-PB could enhance the repair of damaged intestinal epithelial tissue.

**Conclusions:**

Ento-PB demonstrates significant anti-UC activities in OXZ-induced UC rats by regulating the expression levels of inflammatory factors and promoting the repair of colon tissue. This study provides scientific evidence to support the further development of Ento-PB.

## Introduction

Inflammatory bowel disease (IBD) is a group of chronic diseases that cause long-term inflammation of the gastrointestinal tract. The main type of IBD is ulcerative colitis (UC), which affects more than 11.6 per 100,000 people in China^1^. It is characterized by altered immune function and impaired mechanical barrier of the intestinal mucosa^2^^,^ 3. The intestinal mucosa of patients with UC develops expression of interleukin (IL)-4, IL-13, and other Th2-type cytokines, and the level of expression increases as the disease worsens^4^^,^ 5. Neutrophil infiltration of the intestinal mucosa is exacerbated, resulting in a compromised epithelial barrier. Currently, the mainstay of treatment for UC consists of traditional anti-inflammatory agents (glucocorticoids and aminosalicylic acid)^6^ and single-targeted agents (tumor necrosis factor inhibitors) that target the overactive immune response^7^. However, these therapies often produce significant side effects, such as vomiting, generalized edema, and anemia^8^. Therefore, further research into the treatment of UC should focus on the development of multi-targeted, low-toxicity drugs.

Traditional Chinese medicine (TCM) is a system of complementary and alternative medicine that has gained global attention and acceptance in recent years^9^. TCM contains a variety of components with multi-target therapeutic effects. In China, it has shown efficacy in the treatment of glucocorticoid-dependent UC patients who are refractory to aminosalicylic acid^1^^,^ 10. Ento-PB, commonly prescribed in Yunnan, combines both cockroach (*Periplaneta americana* L.) and dandelion (Taraxacum mongolicum Hand.-Mazz.), which have been used in medicine for a long time, and have been documented in China’s first medicinal classic, the Shennong Ben Cao Jing (classic of the materia medica of the divine husbandman)^11^.

A variety of compounds have been found in Ento-PB, such as uracil, hypoxanthine, uridine, adenosine, inosine, caffeic acid, caffeic acid, and chiral acid^12^. Previous studies have shown that Ento-PB from cockroach extract has the potential to ameliorate intestinal damage induced by 1-Chloro-2,4-dinitrobenzene (DNCB) in UC rats and reduce the inflammatory cytokines IL-4, IL-10 and tumor necrosis factor-α (TNF-α)^13^.

Rehabilitation Xin liquid extracted from cockroach extract showed significant relief of localized colonic ulcers in clinical enema therapy^14^. In addition, dandelion enema effectively inhibited the expression of mucosal inflammatory factors^15^. Chen et al.^16^ showed that dandelion extract could alleviate sodium dextran sulfate (DSS)-induced acute colitis in mice by protecting the integrity of the intestinal barrier and reducing the expression of inflammatory factors. It has been reported that Ento-PB modulates the intestinal microbiota and inhibits the over-activation of immune cells in rats with colitis, but the key mechanisms triggering this series of phenomena have not been thoroughly investigated^17^.

Therefore, it is crucial to explore the potential therapeutic mechanism of Ento-PB in the treatment of UC. In this study, we did so by observing the effects of the TLR4/NF-κB signaling pathway on oxazolone (OXZ)-induced UC rats. OXZ-induced models of experimental colitis have several advantages, including a short modeling period, high efficiency, and good reproducibility. The distribution of inflammation, pathologic features, and immunologic characteristics of these models are similar to those of UC patients in the clinically active stage^18^^,^ 19. Therefore, they are commonly used to evaluate the effectiveness of new treatment strategies for UC^20^. Although other studies have confirmed the significant efficacy of Ento-PB, there is no direct evidence of its mechanism of action^21^. The aim of this study was to provide scientific evidence to support the role of Ento-PB in the treatment of UC.

## Methods

### Medicinal materials

Dandelion was purchased from the Dali Traditional Chinese Medicine Market in Yunnan (Yunnan, China), batch number 170306, and was identified as *Taraxacum mongolicum* Hand.-Mazz. by the assistant research fellow Miao He, majoring in pharmacognosy at Dali University. Dried adult cockroach was purchased from Good Doctor Pharmaceutical Co. (Sichuan, China), batch number YF1807014, and was identified as *P. americana* (L.) by professor Zi-zhong Yang, majoring in zoology at Dali University. The research samples were sealed and stored in Yunnan Provincial Key Laboratory of Entomological Biopharmaceutical R&D.

### Preparation of Ento-PB

According to the Yunnan folk Yi nationality prescription, 70 g of cockroach and 30 g of dandelion were weighed, and then 10 times the amount of pure water was added. The mixture was boiled and extracted twice, 2 hours each time. Then, the extract was filtered through gauze. The twice filtrates were combined and concentrated in a rotary evaporator at 50°C to the density of 1.15 g/mL. After that, 95% ethanol was added to the concentrate, which was stirred evenly and stood overnight. The concentrate was then centrifuged at 3,500 rpm/min for 10 min, and the supernatant was taken, concentrated and dried at 50°C to afford the compound Ento-PB extract, which was stored hermetically at -20°C for future use.

### Animals

Sixty SPF grade Sprague-Dawley rats (male and female in half, 6–8 weeks old, 200 ± 20 g) were purchased from Hunan SJA Laboratory Animal Co. [license number: SCXK (Xiang) 2016-0002, Hunnan, China]. The rats were housed in the Experimental Animal Center of Dali University [license number: SYXK (Dian) 2011-0004, Yunnan, China] in an environment with the consistent temperature of 22–24°C, 50–60% relative humidity and a 12-h light-dark cycle. During the experiment, normal laboratory sterilized feed and water were given, and rats were allowed to adapt to the environment for a week. This protocol was approved by the Animal Ethics Committee of Dali University (No. 2018-1006).

### Establishment and evaluation of oxazolone-induced ulcerative colitis model rats

Our models were constructed using the method described by Zhang et al.22 with slight modifications. Briefly, one day before modeling, a 2 cm × 2 cm area on the back of each animal was shaved to expose the skin. Simultaneously, 10 rats (five males and five females) were randomly selected as the normal group. Then, 0.2-mL OXZ (30 mg/mL, anhydrous ethanol) was applied on the exposed area for seven days to induce an allergic reaction. Subsequently, all rats were fasted for 24 hours and then anesthetized with isoflurane. A polyethylene catheter (with the length of 12 cm and the width of 2 mm) was then carefully inserted into the lumen of the colon through the rectum, making the tip of the catheter about 8 cm proximal to the anus. Afterwards, 0.4-mL OXZ (10 mg/mL, 50% ethanol/water mixed solution) was slowly infused into the lumen using a 1-mL syringe via the catheter. After the catheter was slowly removed, the rats were kept in a head-down position for an additional 1 min to ensure distribution of OXZ in the colon. The normal group was operated in parallel according to the above method, but the exposed area on the back was locally smeared with 0.2-mL anhydrous ethanol, and 0.4-mL 0.9% normal saline was infused into the colon.

### Grouping and administration

The remaining rats were randomly divided into six groups (10 rats per group, male and female in half): blank group (Blank), bodel group (Model), sulfasalazine group (SASP; 300 mg/kg), Ento-PB low-dose group (Ento-PB-L, 50 mg/kg), Ento-PB medium-dose group (Ento-PB-M, 100 mg/kg), and Ento-PB high-dose group (Ento-PB-H, 200 mg/kg). The blank group and model group were given 0.9% saline (2.5 mL/kg·d^-1^) by enema. Sulfasalazine group was treated with sulfasalazine suspension (2.5 mL/kg·d^-1^) by enema. Three Ento-PB groups were given Ento-PB solution (2.5 mL/kg·d^-1^) by enema. One day after modeling, all rats were administered once a day. All treatments were carried out for seven days.

### Assessment of disease activity index

Disease activity index (DAI) is a quantized index which was used to estimate the severity of colitis damage. Twenty-four hours after modeling, DAI scores of the model rats were achieved as per the standard established by Hamamoto et al.^23^. The model rats were grouped according to the degree of inflammation: 0–3 points were classified as extremely mild inflammation, 4–6 points were mild inflammation, 7–9 points were moderate inflammation, and 10–12 points were severe inflammation. Rats with extremely mild and mild inflammation were excluded. Therefore, the DAI of each group was evaluated, including weight loss, fecal characteristics, and the degree of bloody diarrhea on the first, fourth, and seventh day of administration.

### Macroscopic assessment of colonic mucosal injury

Twenty-four hours after the last administration, anesthesia of rats was performed by intraperitoneal injection of sodium pentobarbital at 40 mg/kg. The colon tissue was taken and placed on ice to remove mesentery and adipose tissue and dissected longitudinally along the mesenteric side. Then, the colon was cleaned with pre-frozen saline, dried the surface with filter paper, and the weight and length of the colon were recorded. The colonic mucosal damage index (CMDI) was scored according to the criteria described by Lin et al.^24^.

### Histology assessment of colitis

Colon tissues were fixed in 4% paraformaldehyde, embedded in paraﬃn wax, sectioned 5-μm and stained with hematoxylin and eosin (HE). Histological evaluation was performed to assess the intestinal inﬂammation in a blinded fashion by using the scoring system described by Sun et al.^25^. These sections were observed under a microscope at ×20 magnification.

The histological scoring criteria were described as follows:

- Epithelia: normal morphology, 0; lost goblet cells, 1; lost goblet cells in large areas, 2; lost crypts, 3; lost crypts in large areas, 4;
- Inflammatory cell infiltration: no infiltration, 0; infiltrating crypt basis, 1; infiltrating muscularis mucosa, 2; extensively infiltrating muscularis mucosa with thickened mucosa and obvious swelling, 3; infiltrating submucosa, 4.

The total score was calculated based on addition of the two scores.

### Detection of serum tNOS, iNOS activities and EGF, IL-13, IL-4, IL-10 levels

At the end of the experiment, all rats were anesthetized by intraperitoneal injection of 10% chloral hydrate. Blood was collected from the abdominal aorta. The serum was separated by centrifugation at room temperature of 3,500 rpm/min for 10 minutes and stored at -80°C for further detection. According to the manufacturer’s instructions, the contents of inducible nitric oxide synthase (iNOS), total nitric oxide synthase (tNOS), interleukin-4 (IL-4), interleukin-10 (IL-10), interleukin-13 (IL-13), and epidermal growth factor (EGF) in rat serum was measured by enzyme-linke immunosorbent assay (ELISA) kits.

### Detection of MPO activity and inflammatory cytokines TNF-α level in colon tissue

Colon segments were homogenized with phosphate buffer saline (1:9, w/v) and then centrifuged for 10 min (3,500 × g, 4°C). Supernatant was collected for detection of activities of colonic myeloperoxidase (MPO) and levels of inflammatory cytokines TNF-α with ELISA kits following manufacturer’s instructions.

### Western blot analysis

Frozen colon tissue samples were homogenized in ice-cold RIPA buffer with a cocktail of protease inhibitors. The homogenate was then kept on ice for 30 minutes and centrifuged at 12,000 rpm for 10 minutes at 4°C. The supernatant was then analyzed for protein concentration by using the BCA protein assay kit. The protein concentration of the supernatant was quantified using the BCA protein assay kit. After mixing with the supernatant, the supernatant was boiled in protein upload buffer at 100°C for 10 minutes, then an equal amount of protein was separated by 10% SDSPAGE and transferred to a 0.45-µM polyvinylidene difluoride (PVDF) membrane. The membranes were blocked with triple buffered saline (TBST) containing 5% skimmed milk for 1 hour at room temperature. After washing the membrane three times with TBST, the membrane was incubated with primary antibody at 4°C overnight.

The following primary antibodies were used in the experiments: β-actin (1:2,000), NF-κB p65 (1:1,000), p-NF-κBp65 (1:600), MYD88 (1:1,000), p-MYD88 (1:600), TLR4 (1:1,000) and p-TLR4 (1:600). After four washes in TBST buffer for 5 min each, the cells were incubated with the secondary antibody (goat anti-rabbit IgG-HRP, 1:3,000 dilution) for 1 h at room temperature. Finally, protein bands were detected using a chemiluminescence detection system with enhanced chemiluminescence (ECL) substrate washed five times in TBST buffer for 5 min each. The integration density of each relevant protein band was calculated using ImageJ, and protein levels were normalized to β-actin levels.

### Statistical analysis

Experimental data are represented as the mean ± standard deviation, and statistical analyses were performed by using Statistical Package for the Social Sciences 24.0^26^. One-way analysis of variance was used for comparisons among multiple groups. Least significant difference (LSD)-t test was used to compare the data with the same variance between the two groups. If the variance was heterogenous, Tamhane’s t-test was performed. *p* < 0.05 was considered statistically significant.

## Results

### Ento-PB improves the symptoms of oxazolone-induced acute colitis in rats

**Figure 1:**
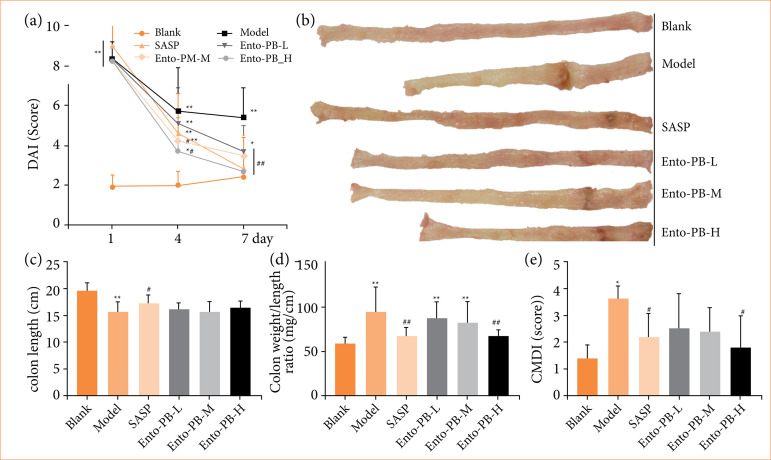
Ento-PB alleviated clinical symptoms in oxazolone-induced colitis. **(a)** Disease activity index; **(b)** representative colon pictures; **(c)** colon length; **(d)** colon weight/length ratio; **(e)** colon mucosal damage index. Quantitative data are shown as the mean ± standard deviation, n = 10.

The DAI is one of the important indicators of disease severity and drug efficacy in clinical practice^27^. Decreased DAI scores indicate the remission of inflammation in UC patients. After modeling, as shown in Fig. 1a, the DAI score of rats treated with OXZ was significantly higher than that of the blank group (all *p* < 0.01), indicating that the modeling was successful. After SASP and Ento-PB treatment, the DAI score was gradually decreased. At the last administration, the DAI score of the model group was still higher than that of the blank group, SASP group, and Ento-PB group (*p* < 0.01). The DAI scores of the SASP group and Ento-PB-H group were similar to that of the blank group (*p* > 0.05).

SASP: sulfasalazine; *p < 0.05; **p < 0.01 vs. blank group; #*p* < 0.05; ##*p* < 0.01 vs. model group. Source: Elaborated by the authors.

The colon length and colon weight/length ratio are indexes of colitis severity^28^. As shown in Figs. 1 and 1d, OXZ-induced colitis may lead to shortened colons and an increased colon weight/length ratio. However, SASP and Ento-PB treatment could hinder the colon from shortening and reduce the colon weight/length ratio. The efficacy of Ento-PB-H was close to SASP. Moreover, as shown in Figs. 1b and 1d, the CMDI score of the model group was significantly higher than that of the blank group (*p* < 0.01), Ento-PB-H group and SASP group (*p* < 0.01 or *p* < 0.05), while the Ento-PB-L and Ento-PB-M groups had no significant improvement in CMDI scores.

### Ento-PB reduces pathological damage of colon tissue in ulcerative colitis rats

**Figure 2:**
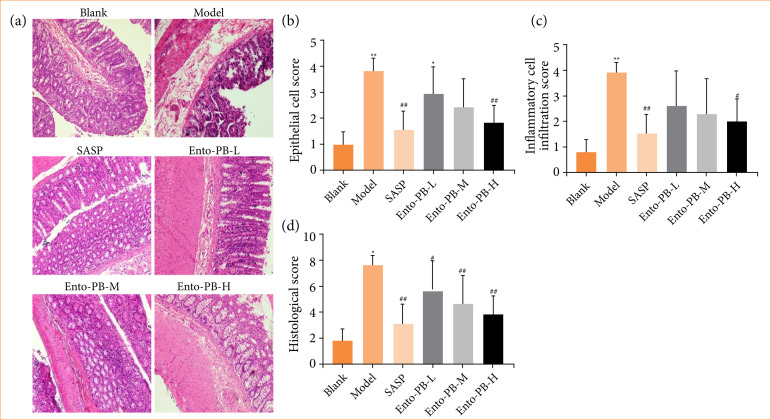
Ento-PB alleviated pathological alterations in oxazolone-induced colitis (x200). **(a)** Representative images of hematoxylin and eosin-stained colon tissue; **(b)** epithelial cell injury was assessed by analysis of the hematoxylin and eosinstaining; **(c)** infammatory cell infltration was assessed by analysis of the hematoxylin and eosin staining; **(d)** histological score. Quantitative data are shown as the mean ± standard deviation, n = 10.

Compared with the blank group (Figs. 2a–2c), the model group showed a loss of massive goblet cells, crypt disappearance, mucosal necrosis, lamina propria hemorrhage, mucosal muscle edema, and inflammatory cell infiltration into the mucosa or submucosa. After SASP and Ento-PB treatment (Figs. 2a–2d), histopathology scores were significantly reduced (both *p* < 0.05). The administration of Ento-PB-H significantly improved the colonic tissue lesions induced by OXZ. The improved colonic mucosa hyperemia decreased infiltrating inflammatory cells, and the relatively intact colonic mucosa structure and crypt structure confirmed the protective effect of Ento-PB.

SASP: sulfasalazine; *p < 0.05; **p < 0.01 vs. blank group; #*p* < 0.05; ##*p* < 0.01 vs. model group. Source: Elaborated by the authors.

### Effect of Ento-PB on serum iNOS, tNOS activity and IL-4, IL-10, IL-13, and EGF levels in ulcerative colitis rats

**Figure 3:**
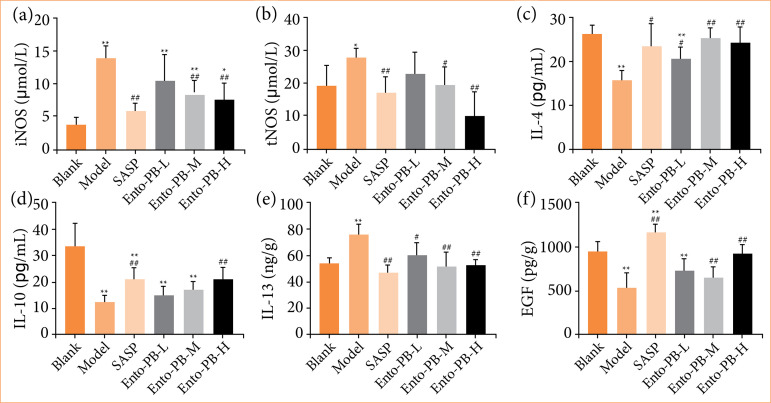
Effects of Ento-PB treatment on the expression of serum inflammatory mediators after oxazolone-induced ulcerative colitis. (a–f): the expression levels of iNOS, tNOS, IL-4, IL-10, IL-13, EGF in serum were measured by enzyme-linked immunosorbent assay analysis. Quantitative data are shown as the mean ± standard deviation, n = 10.

The increase of iNOS level can cause the release of a large amount of nitric oxide (NO), which in turn promotes the release of a large number of inflammatory cytokines, and causes tissue damage, which exacerbates the progress of UC. As shown in Figs. 3a and 3b, compared with the model group, SASP and Ento-PB could significantly inhibit the expression levels of iNOS and tNOS in serum (*p* < 0.05 or *p* < 0.01). As shown in Figs. 3c–3e, compared with the blank group, the proinflammatory markers (IL-13) in the serum of the model group were significantly increased (*p* < 0.01), and the anti-inflammatory markers (IL-4, IL-10) significantly reduced (*p* < 0.01). After SASP and Ento-PB-H treatment, the expression of IL-13 was significantly inhibited (*p* < 0.05), while the expression of IL-4 and IL-10 increased (*p* < 0.05).

SASP: sulfasalazine; *p < 0.05; **p < 0.01 vs. blank group; #*p* < 0.05; ##*p* < 0.01 vs. model group; iNOS: inducible nitric oxide synthase; tNOS: total nitric oxide synthase; IL: interleukin; EGF: epidermal growth factor. Source: Elaborated by the authors.

EGF relieves mucosal inflammation by promoting the proliferation of gastrointestinal epithelial cells, preventing excessive production of proinflammatory mediators and reducing epithelial cell apoptosis29. In this study (Fig. 3f), after treatment with SASP and Ento-PB, the level of EGF in rats increased significantly (p < 0.01), which was close to the blank group (p > 0.05).

### Effects of Ento-PB on MPO activity and TNF-α level in colon tissue of ulcerative colitis rats

**Figure 4:**
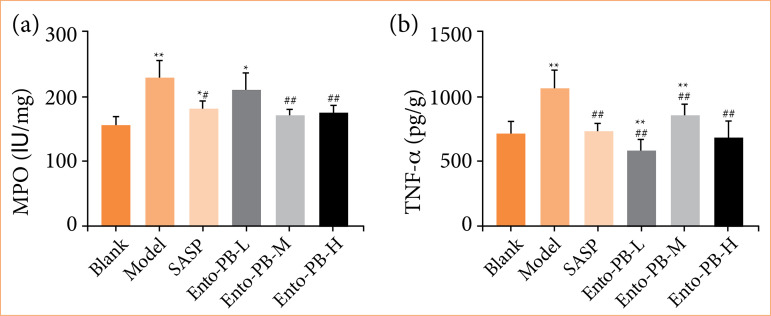
Effects of Ento-PB treatment on the expression of colonic inflammatory mediators after oxazolone-induced ulcerative colitis. (**a** and **b**) The expression levels of MPO and TNF-α in colon tissue were measured by enzyme-linked immunosorbent assay analysis. Quantitative data are shown as the mean ± standard deviation, n = 10.

MPO is an enzyme found primarily in neutrophils and has been used as a quantitative indicator of inﬂammation and neutrophil inﬁltration in the colonic mucosa^30^. The decrease of MPO activity could be interpreted as the performance of the anti-inﬂammatory properties. In this study, as shown in Fig. 4a, compared with the blank group, the content of MPO in colon tissue of the model group was significantly increased (*p* < 0.01). Compared with the model group, the content of MPO in colon tissue of rats was significantly decreased (*p* < 0.05 or *p* < 0.01) after SASP, Ento-PB-M, and Ento-PB-H treatment. TNF-α is an important proinflammatory cytokine. Excessive TNF-α can lead to the destruction of cells and the release of inflammatory mediators^31^. It is considered to be the determinant of the amplification of UC mucosal inflammation.

SASP: sulfasalazine; *p < 0.05; **p < 0.01 vs. blank group; #*p* < 0.05; ##*p* < 0.01 vs. model group; MPO: myeloperoxidase; TNF: tumor necrosis factor-α. Source: Elaborated by the authors.

As shown in Fig. 4b, compared with the blank group, the level of TNF-α of the model group was significantly increased (*p* < 0.01). After treatment with SASP and Ento-PB, it was able to significantly inhibit the increase of TNF-α level (*p* < 0.01). It is worth noticing that the expressions of MPO and TNF-α in the colon of rats in the Ento-PB-H group had almost returned to normal levels (*p* > 0.05).

### Effect of Ento-PB on the regulation of key molecules of the NF-κB with TLR4 and MyD88 activities in a ulcerative colitis model

**Figure 5:**
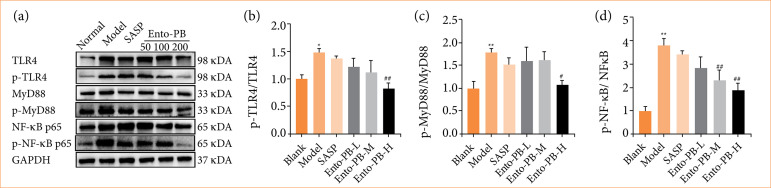
The effect of extracts of dandelion and American cockroach (Ento-PB) on the levels of key molecules involved in the nuclear factor-kappa-B (NF-κB) signaling pathway, colonic toll-like receptor 4 (TLR4) and myeloid differentiation factor 88 (MyD88) NF-κB phosphorylated p-65 expression. Data are expressed as mean ± standard deviation.

The western blotting method was used to detect NF-PB-p65 and p-NF-KB-p65 protein levels in the colon. As shown in Figs. 5a–5d, we found that p-p65 protein expression was increased in the model group compared with the blank group (*p* < 0.01). In contrast, p-p65 expression in the Ento-PB-H treatment group was significantly lower than that in the Ento-PB-L treatment group (*p* < 0.01). Meanwhile, Ento-PB reduced the level of p-p65 in a dose-dependent manner. TLR4/MyD88 signaling pathway plays an important role in inflammation induction by regulating the activity of transcription factors.

SASP: sulfasalazine; *p < 0.05; **p < 0.01 vs. blank group; #p < 0.05; ##p < 0.01 vs. model group. Source: Elaborated by the authors.

In addition, the results showed that the expression of TLR4 and MYD88 was significantly up-regulated in both epithelial and inflammatory cells of colon tissue in the model group compared to the control group. The expression of TLR4 in colon tissues was significantly inhibited when Ento-PB was administered. The inhibitory effect of the high dose was better than that of the low dose.

## Discussion

Nucleosides have been proven to be one of the main active components in both the drug “Kangfuxin liquid” (made from cockroach extract), for clinical gastrointestinal ulcer treatment, and the drug “Xinmailong injection” (extracted and purified from cockroach), for cardiovascular disease treatment in China^32^. Studies have established that caffeic acid exhibits significant antioxidant, antibacterial, and anti-inflammatory effects in the clinical treatment of leukopenia^33^^,^ 34. Caftaric acid plays an anti-oxidant and anti-inflammatory role in the treatment of indomethacin-induced gastric ulcers in rats^28^. Xue et al.^35^ have observed that chicoric acid in dandelion can alleviate lipopolysaccharide-induced inflammation in intestinal epithelial cells by regulating the inflammatory signal NF-κB p65 and the expression levels of inflammatory factors such as COX-2 and IL-1β. The identification of the aforementioned components directly or indirectly provides evidence for the effectiveness of Ento-PB in the treatment of UC.

The results of this study demonstrated that Ento-PB and SASP significantly reduced the DAI score and CMDI score of rats with colitis. Additionally, they prevented the colon from shortening and decreased the colon weight/length ratio. The epithelial detachment and inflammatory infiltration of the colonic mucosae and submucosae were relieved after treatment with Ento-PB. The histopathology scores of the rats treated with Ento-PB were lower than those of the rats in the model group.

It is noteworthy that Ento-PB-H had a greater improvement effect than SASP. Upon the onset of UC, polymorphonuclear leukocytes, predominantly neutrophils, are recruited to the intestinal wall by NO. This recruitment promotes the release of various inflammatory mediators, disrupts the stability of cell membranes, and induces apoptosis^36^. Ento-PB could reverse the infiltration of inflammatory cells into the colon tissue of UC rats by downregulating the expression of serum iNOS and tNOS. In addition, the local inflammation level and the expression of MPO in colon tissue were also reduced, which was consistent with the results of histopathological examination.

Excessive inflammatory immune responses are associated with UC lesions and relapse. OXZ induces CD4^+^ T-cell-mediated delayed hypersensitivity in the intestinal mucosa tissue, causing a shift in the Th1/Th2 ratio towards Th2 and the pathogenic activation of Th2 immune cells. This, in turn, promotes the development of UC^37^^,^ 38. The increased secretion of IL-4 initially plays a dominant role in driving the development of inflammation, but it is quickly replaced by IL-13^39^. Although IL-4 and IL-13 are structurally analogous and share functional receptors, their functions are quite different^40^. IL-4 is a crucial cytokine that is skewed towards Th2 cells and possesses potent anti-inflammatory properties. It can inhibit the production of TNF-α and IL-1β by mononuclear macrophages^39^. IL-13 acts as an effector cytokine and can initiate epithelial and fibrotic reactions^40^.

Excessively high concentrations of IL-13 in local areas can hinder the expression of EGF and tight junction proteins, thereby compromising the integrity of colonic epithelial tissue.

In addition, the decreased expression of factors that regulate Th0 polarization in OXZ-induced colitis rats leads to the accumulation of a significant number of Th1 cells in the intestinal mucosa and a relative absence of Th2 cells^41^. Therefore, hypersecretion of TNF-α and hyposecretion of IL-10 occur. IL-10 is a prototypical anti-inflammatory and immunosuppressive Th2-type cytokine^42^. It functions by inhibiting the expression of TNF-α, IL-2, and other pro-inflammatory factors at the molecular level. Additionally, it downregulates the level of differentiated Th1 cells^42^.

Elevated TNF-α levels in the colonic mucosa can activate various inflammatory signals, including iNOS and NF-κB pathways^43^. This activation can lead to a “waterfall-style” inflammatory cascade and plays a role in positive feedback regulation, ultimately causing persistent damage to the intestinal mucosal tissue^43^. Therefore, it is essential to alleviate UC by regulating the levels of inflammatory cytokines. In this study, after administering Ento-PB to rats with OXZ-induced colitis, the expression levels of IL-13 and TNF-α significantly decreased, while the expression levels of IL-4, IL-10, and EGF sharply increased.

TLR4 plays a key role in the inflammatory process^44^. Numerous studies have shown that TLR4 is overexpressed in the colonic mucosa of patients with colorectal cancer^45^. However, TLR4 knockout mice have shown a significant reduction in the incidence of colon cancer^46^. The activation process of TLR4/NF-κB/MAPKs involves several crucial steps. MyD88 is an important downstream component of the TLR4 signaling pathway. Therefore, we examined the expression of TLR4 and MyD88 proteins in the colon using western blotting.

The results showed that the TLR4 protein level was highly expressed in the model group of mice, while it was significantly reduced in the Ento-PB-H treatment group compared to the Ento-PB-L group. Notably, there was a significant difference in MyD88 protein expression levels between the control and model groups, suggesting that Ento-PB may play an important role through the TLR4/NF-κB signaling pathway. The present study demonstrated that Ento-PB has significant anti-UC activity in OXZ-induced UC rats, and it can regulate the expression level of inflammatory factors and promote the repair of colonic tissues, which has a broad application prospect, and the present study provides a data reference and scientific basis for the further development of Ento-PB in the future.

However, this paper still has some limitations. First, although Ento-PB is rich in nucleoside ingredients and phenolic acids, further research is needed to determine whether they are the components responsible for the UC-alleviating effect. Second, we only evaluated the effect of Ento-PB on inflammatory factors and repair factors in UC rats. However, the anti-inflammatory mechanism is still unclear and requires further exploration.

## Conclusion

In summary, Ento-PB prevents colon shortening and decreases colon weight/length ratio. Improving colonic mucosal congestion and reducing inflammatory cell infiltration significantly inhibit serum expression of iNOS and tNOS, and expression of IL-13, increase expression of IL-4 and IL-10, elevate EGF levels, reduce MPO content in colonic tissues, inhibit elevated levels of TNF-α, and reduce p-p65 expression TLR4 expression. Our findings demonstrate, for the first time, that the administration of Ento-PB effectively reduces colon inflammation in rats.

## Data Availability

All dataset were generated or analyzed in the current study.
